# Cocain and Its Many Uses in Dentistry

**Published:** 1902-10-15

**Authors:** Elliott R. Carpenter

**Affiliations:** Chicago


### COCAIN AND ITS MANY USES IN DENTISTRY.

BY ELLIOTT R. CARPENTER. D.D.S., CHICAGO.

About six years ago Dr. C. Gurnee Fellows, the rhinologist of this city, called my attention to the powdered crystals of hydrochlorate of cocain as a quick and safe anesthetic for operations upon the mucous membrane, and I first used it in excising the flap of gum overgrowing the distal half of the crown of a lower third molar, removing same with but little pain to my patient.

It then occurred to me that it might be a good thing to use upon the gum tissue where it was necessary to crowd the gum away in applying clamps to buccal and labial gingival cavities, but I did not meet with a great degree of success, and it was several weeks before I discovered the cause of my failure, the reason being that I had not anesthetized the poricementum. Now I pack my powdered crystals of cocain under the free margin of the gum against the pericementum at points where both jaws of the clamp grasp the root, and in a minute to a minute and a half I can press the gum away from the margin of the cavity, and force the clamp to place with but little or almost no pain to the patient. In this procedure I will add that the rubber dam is applied first, and the hole of the rubber dam encircling the tooth to be thus anesthetized is drawn to one side by the forefinger, enlarging it just enough to expose the edge of the gum, when cocain can be readily packed between gum and pericementum without the danger of getting it into the mouth and having the saliva carry it to the tongue and throat. Should the cavity be so far beneath the gum as to preclude the possibility of forcing the gum out of the way, I unhesitatingly clamp well on the gum itself, and dissect away the exposed flap with a sharp spoon excavator, dipping same into a 95 percent solution of carbolic acid before using. The acid sears over the edge of wound, thus inhibiting the action of microörganisms upon the freshly cut surfaces after the clamp has been removed. Furthermore, I am convinced after six years of close observation in daily practice that in thus cocainizing the peridental membrane the effect of the anesthesia is, to a considerable degree, carried to the pulp of the tooth. Since I have taken special precaution to anesthetize the peridental membrane, I observe that in fully nine-tenths of such cavities I can excavate with much less pain than ever before, and in many cases without pain at all. Thus you have the results of my clinical experience.

I am not a good theorist, but I have sought hard for the truth, and believe after an earnest study of Professor Black's "Peridental Membrane," especially Chapter IX, touching upon the blood supply of the peridental membrane, that I have a possible solution of cause and effect. Professor Black says: "The large arteries enter the alveolus mostly at the apical space, or, rather, one or two vessels enter here, and immediately break up into smaller ones. One or two of these enter the root canal to supply the pulp of the tooth, while others, four, six or eight, pass down the sides of the root to supply the peridental membrane. In their passage down the membrane these divide into many branches; some vessels, however, are continuous from the apical space to the gingivus.

I believe firmly that this anesthesia is carried to the pulp through the medium of the few blood vessels in the membrane that are continuous from the apical space to the gingivus.

Pulp extirpation under cocain is one of the best demonstrations of what a boon this drug is to the dental profession, and inasmuch as this special operation was so well described by Dr. R. C. Young, of Anniston, Alabama, in an article published in the January *Cosmos*, of 1900, I will not go into a detailed description of it here. The only point in which I would differ with Dr. Young is that he moistens a piece of punk and dips same into cocain crystals, and I make a saturated solution from the ground crystals, in which I soak a few shreds of cotton, and apply to the exposure before placing the soft rubber over for the pumping process, thereby avoiding irritation to the pulp from small particles of the crystals that are not always dissolved by the water. In freshly exposed pulps a minute to a minute and a half is sufficient time to produce complete anesthesia, when the exposure can be enlarged and the pulp extirpated. I have had good success with the saturated solution of cocain applied to inflamed pulps, except where the inflammation was of long standing, and had become chronic. Arsenic would here be contra-indicated, so the poor old drug that has served us faithfully for so many years will, I believe ultimately be relegated with the turnkey to oblivion. Aside from the matter of saving time and pain, teeth having pulps extirpated under cocain retain their normal color to a much greater degree than where arsenic is employed for the same operation.

The saturated solution of cocain has now become a daily necessity in my practice, as I use it always in fitting bands for crowns, adjusting separators, inserting wedges, ligating the rubber dam (when this procedure is unavoidable), and in excising hypertrophied gum tissue.—*Review*.

